# Effect of a 16-Week Exercise Program After Bariatric Surgery on Sarcopenia Parameters Based on FNIH, EWGSOP2, and EASO/ESPEN Criteria: the Results of the EXPOBAR Randomized Trial Program

**DOI:** 10.1007/s11695-025-08142-7

**Published:** 2025-08-13

**Authors:** Cláudia Mendes, Manuel Carvalho, Carolina A. Cabo, Jorge Bravo, Sandra Martins, Armando Raimundo

**Affiliations:** 1 Universidade de Évora, Comprehensive Health Research Centre (CHRC), Évora, Portugal; 2 CBIOS - Universidade Lusófona’s Research Center for Biosciences & Health Technology, Lisboa, Portugal; 3 Unidade Local Saúde Alentejo Central - Hospital Espírito Santo de Évora, EPE, Évora, Portugal; 4 Instituto Politecnico de Setubal, Setúbal, Portugal; 5 Universidade de Évora, Escola de Saúde e Desenvolvimento Humano, Departamento de Desporto e Saúde, Évora, Portugal; 6 Research Center in Sports Sciences, Health Sciences and Human Development, CIDESD, Vila Real, Portugal; 7 CIDESD, Universidade Europeia, Lisbon, Portugal

**Keywords:** Exercise, Bariatric surgery, Fat-free mass, Sarcopenia, Skeletal muscle mass

## Abstract

**Background:**

Bariatric surgery is a recognized treatment option for severe obesity, and its effectiveness in reducing weight and controlling obesity-related conditions has been demonstrated. However, it can also lead to decreased skeletal muscle mass and strength, increasing the risk of sarcopenia after surgery. This randomized clinical trial studied the effects of a 16-week exercise program on sarcopenia in bariatric surgery patients.

**Methods:**

Thirty-seven surgery candidates participated in the EXPOBAR (EXercise POst BARiatric) program and were randomized into experimental or control groups. The intervention lasted 16 weeks, starting 1 month after surgery, and included a circuit training with aerobic and resistance exercise intervention. The outcomes included physical fitness parameters (anthropometry, body composition, and physical performance) and were assessed at four time points. All participants underwent gastric bypass surgery (RYGB).

**Results:**

The EXPOBAR trial revealed significant and meaningful effects of the exercise intervention on anthropometric indices, such as weight (*p* = 0.039) and waist circumference (*p* = 0.010). The most substantial improvements were observed in physical function and strength metrics (*p* = 0.005 and *p* < 0.001, respectively), along with a reduction in fat mass (*p* = 0.006), indicating the intervention’s effectiveness on sarcopenia parameters and in enhancing both physical fitness and body composition.

**Discussion:**

Current findings indicate that following an initial decrease due to bariatric surgery, exercise training significantly improves functional physical capacity and strength. The exercise program in this study effectively reversed the surgery-induced loss in function and strength, reducing the number of patients at risk of sarcopenia. Physical and functional capacity are crucial noninvasive indicators for diagnosing muscle quality and sarcopenia.

**Conclusion:**

Long-term management of sarcopenia and sarcopenic obesity in bariatric surgery patients requires frequent monitoring of body composition and muscle function. This approach is essential for tracking progress and optimizing treatment strategies over time. This study highlights the importance of integrating structured exercise programs into after bariatric surgery care to mitigate the risk of sarcopenia.

**Trial Registration:** The trial was registered at Clinicaltrials.gov (NCT05289219).

## Introduction

Obesity is a significant public health issue because it is closely linked to increased mortality and morbidity rates. It is a chronic illness and is related to other chronic conditions, such as metabolic syndrome, diabetes, cardiovascular disease, psychological disorders, and social problems [1].

Surgical treatment is considered the most effective management tool for morbid obesity and is known for its ability to induce substantial weight loss (60% or more excess weight) and improve associated obesity complications. However, the weight loss achieved after bariatric surgery is not only due to fat reduction but also due to a significant loss of lean tissue, accounting for up to 25% of the overall body mass reduction [2–4]. This reduction in lean tissue mass, especially muscle mass, poses a challenge because it can decrease both resting and activity-related energy expenditure.

Healing after major surgical procedures can cause a significant increase in the body’s need for protein. However, in the initial months following bariatric surgery, there is typically a significant decrease in food intake [5]. This can result in temporary protein deficiency, especially after surgical interventions, which can affect the body’s ability to absorb nutrients. This deficiency usually resolves once the patient’s metabolism adjusts to the changes caused by surgery and food intake becomes sufficient and consistent according to needs [6–8].

The muscle requires energy to function properly, so a decrease in muscle mass can decrease both resting and activity-related energy expenditure. As a result, patients with obesity and sarcopenia or at risk of sarcopenia before surgery are less likely to lose weight and are more prone to regain weight than those who have a healthy skeletal muscle system [9]. After surgery, the combination of muscle tissue loss, temporary protein deficiency, and surgical trauma can contribute to or worsen preexisting conditions of sarcopenia [10].

Furthermore, if muscle deficiency occurs or worsens after surgery, weight loss may slow or stop altogether, increasing the likelihood of obesity relapse. Additionally, sarcopenia increases the risk of metabolic complications and can ultimately compromise life expectancy, even if weight loss is achieved [11, 12].

For bariatric patients with severe obesity, muscle deficiency can give rise to notable clinical complications following surgery. Preoperative sarcopenia has been shown to be a reliable indicator of complications and mortality during major abdominal surgery. Additionally, older individuals face an increased risk of cardiovascular events in the perioperative period. It is worth noting that the age of patients seeking metabolic/bariatric surgery may increase, leading to a greater number of individuals older than 60 years undergoing the procedure, which is when sarcopenia becomes a clinically significant issue [13]. Therefore, it is crucial to carefully consider the occurrence of complications in patients with obesity and sarcopenia who undergo significant surgical bariatric procedures [14].

The European Working Group on Sarcopenia in Older People (EWGSOP) established a structured approach to identify, evaluate, and address issues related to sarcopenia in 2010. In 2019, they published a consensus based on the presence of low lean mass and low muscle strength, the EWGSOP2. This document uses the Find-Assess-Confirm-Severity (F-A-C-S) algorithm, which is utilized in various fields, including medicine, psychology, and risk management [15, 16].

Furthermore, in 2014, the Foundation of the National Institutes of Health (FNIH) published a standardized diagnostic approach with the Sarcopenia Project, which suggested adjusting the criteria to account for differences in body mass index (BMI) [17]. BMI is a crucial criterion for selecting patients for obesity surgery [18]. Given that few studies have assessed sarcopenia and sarcopenic obesity after bariatric surgery, additional experimental studies are needed so that clear criteria can be defined regarding useful tools and procedures to prevent and treat sarcopenic obesity after bariatric surgery.

More recently, in 2022, the European Association for the Study of Obesity (EASO) and the European Society for Clinical Nutrition and Metabolism (ESPEN) recommended introducing weight adjustment, particularly for individuals with obesity who are at risk of developing sarcopenia [19], considering that sarcopenia and muscle mass loss are significant concerns for individuals undergoing bariatric surgery.

Several guidelines suggest a combination of moderate-intensity exercise to maintain muscle mass. The American College of Sports Medicine (ACSM) recommends a progressive exercise program for all individuals following the FITT-VP principle, which includes frequency, intensity, time, type, volume, and progression [20, 21]. Similarly, the American Society for Metabolic and Bariatric Surgery (ASMBS) advocates starting a progressive walking program on the first day after surgery, incorporating aerobic exercises and strength training for at least 30 min daily [22, 23].

Regular and targeted exercise programs are fundamental for managing sarcopenia and sarcopenic obesity. Resistance training has been proven effective at promoting muscle strength, enhancing function and improving body composition by reducing excess adiposity [24–26].

The combination of strength training and aerobic training has been shown to improve strength and metabolism in individuals with obesity. However, the long-term incidence of sarcopenia after surgery remains unclear, underscoring the need for research on both short-term and long-term exercise programs.

## Objectives

The primary objective was to analyze the effects of a 16-week exercise program on sarcopenia parameters in patients undergoing RYGP. Secondary outcomes included body composition metrics and functional capacity measures, while exploratory outcomes included quality-of-life assessments and diagnostic criteria.

## Methods

### Study Design

This randomized controlled study (NCT05289219) was developed at a single health institution, the Center for Integrated Responsibility of Bariatric Surgery and Metabolic Diseases (CRI. COM), performed at a Portugal Hospital (ULSAC) and at the University (ESDH-CHRC). The protocol has been previously described [27].

Recruitment took place between December 2021 and December 2023 from among candidates who met the diagnostic criteria for bariatric surgery. A team member of the two institutions managed all the procedures.

A bariatric surgeon and a sports specialist nurse contacted the patients and randomized them into the control group (CG) and intervention group (IG). The invitation to participate was made in the context of the outpatient office, and participants who agreed to participate in the study were given the free and informed consent form previously approved by both the University and Hospital Ethics Committee (HESE_CE_1917/21).

**Fig. 1:**
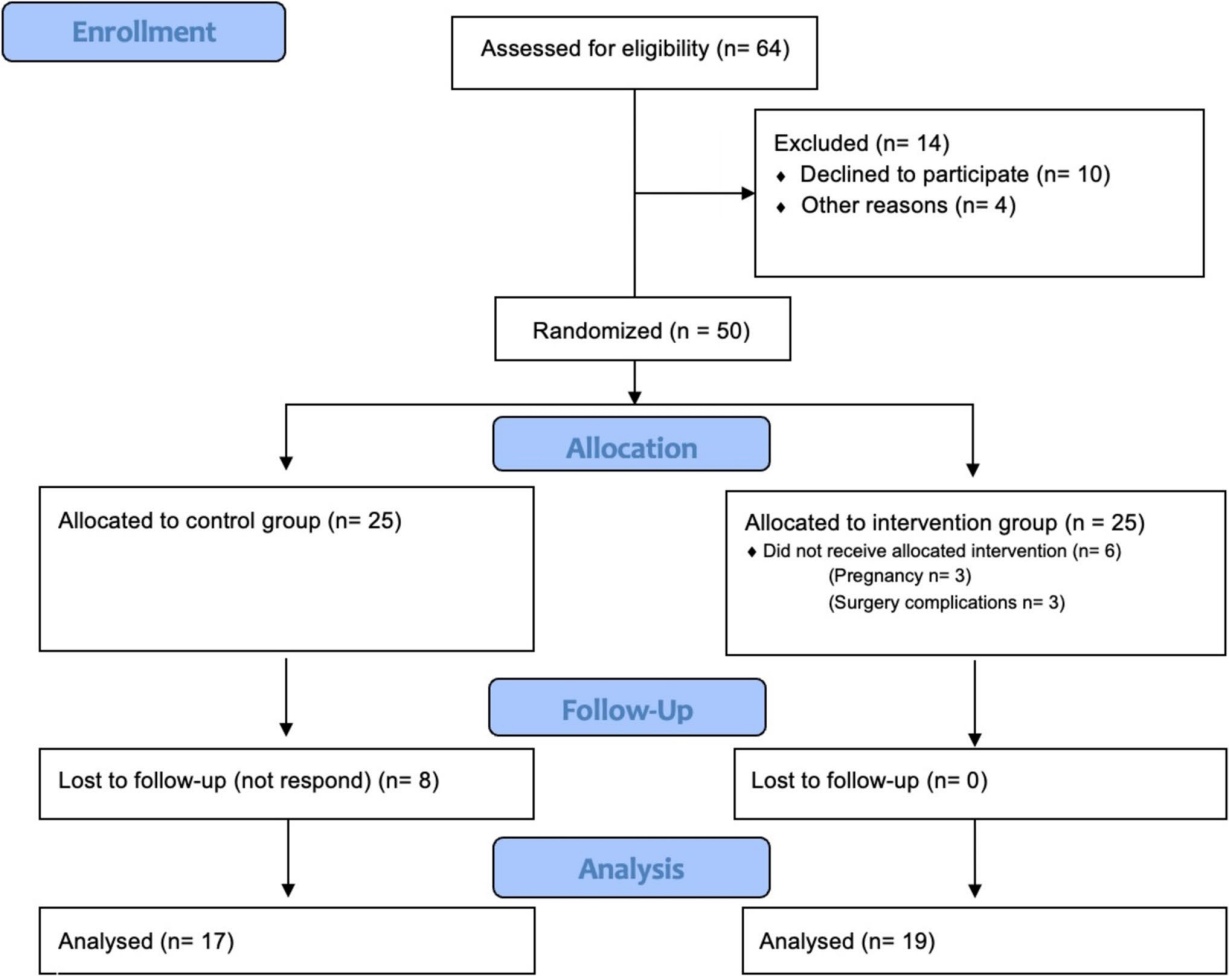
Consort flow diagram

Exercise training began 1 month after surgery, with a frequency of three times per week, up to a maximum of 55 min per session, for 16 weeks. The study included four evaluations over a 17-month period. All assessments were conducted by researchers who were blinded to the study’s objectives and the participants’ group allocation to minimize potential biases and ensure the integrity of the data collected. This study protocol complied with the CONSORT 2010 recommendations (Fig. 1).

### Eligibility Criteria

The inclusion criteria for patients were as follows: were candidates for bariatric surgery, aged between 18 and 60 years, had a BMI between 35 and 50 kg/m^2^, were men and women, had no contraindications to exercise, and agreed to participate voluntarily in the study. The BMI range was chosen to target individuals with moderate to severe obesity. Patients with other previous bariatric surgical interventions or bariatric surgery complications were excluded from the study.

### Sample Size and Randomization

This was a prospective study, and the sample size was calculated by the G*power [28]. A total of 17 participants were included in each group to enable the detection of a moderate estimated effect size (between-group differences) of at least 0.99 standard deviations in the outcome risk of sarcopenia [29, 30]. Two-way independent sample *t*-tests were performed with an alpha error of *α* = 0.05 and a power of 1 − *β* = 0.80.

Patients proposed for bariatric surgery (gastric bypass-RYGB) were randomly assigned at the time of proposal by a systematic random process to usual care (CG) or usual care plus an exercise program (IG).

### Outcomes

The evaluations are made in relation to the exercise program. After surgery, all outcomes were assessed before and after the exercise program. The first assessments were performed before the training program (T0—pre-exercise—1 month after surgery), and the remaining three were performed at different moments after the exercise program (T1—after-exercise—5 months after surgery; T2—6 months after-exercise—11 months after surgery; and T3—12 months after-exercise—17 months after surgery).

#### Anthropometry and Body Composition

Weight was measured with a digital scale (Tanita MC 780-P MA), and height was determined by a manual stadiometer. This assessment was made before breakfast and at least 6 h after eating, without shoes or wearing light clothes. Waist circumference was determined using a measuring tape based on the International Society for the Advancement of Kinanthropometry (ISAK) protocol [31–33], and BMI was calculated (weight/height^2^) [34, 35]. Dual-energy X-ray absorptiometry (DEXA) (DXA, Hologic QDR, Hologic, Inc., Bedford, MA, USA) was used to estimate body composition (fat mass and skeletal muscle mass) [30].

##### Sarcopenia Screening—FIND

In clinical practice, the EWGSOP2 and EASO/ESPEN both recommend the use of the SARC-F for sarcopenia screening and assessment [15, 16, 36]. All participants completed this tool at the four time points.

This tool consists of five questions: strength (S), assistance walking (A), rising from a chair (R), climbing stairs (C), and falling (F). A total score is calculated out of 10 for each question, ranging from “not at all” to “very difficult.” The recommended cutoff value for predicting sarcopenia is ≥ 4 points. Low values indicate no risk of sarcopenia [37–39].

#### Sarcopenia Diagnosis—ASSESS

##### Skeletal Muscle Strength:

Sarcopenia is primarily diagnosed by searching for weak muscles. To assess evidence of sarcopenia, the EWGSOP2 recommends the use of grip strength or a chair stand measure with specific cutoff points for each test.

The handgrip strength test was performed using manual pressure dynamometry (Jamar®) to assess the muscle strength of the upper limbs. The participants were told to stand with their elbows straight and completely relaxed. The muscle strength test value was determined by recording the greatest grip strength value obtained after two trials with each hand [40, 41].

The sit-to-stand test, which requires participants to alternate between standing and sitting for 30 s as often as they could, was used to assess the muscle strength of the lower limbs [42]. The chair stand test provides a trustworthy and useful way to measure strength because it assesses both endurance and strength [43, 44].

A handgrip strength of less than 27 kg for men and less than 16 kg for women [45] or > 15 s for five rises on the chair rise test (5-times sit-to-stand) were the chosen cut-offs. The timed chair stand test is a variation that counts how many times a patient can rise and sit in the chair over a 30-s interval [46].

#### Sarcopenia Confirmation – CONFIRM

##### Skeletal Muscle Mass:

Different methods can be used to report skeletal muscle mass, such as dual-energy X-ray absorptiometry (DEXA), bioelectrical impedance analysis (BIA), or muscle cross-sectional area (MRI) analysis. Given that DEXA is a widely used technique for determining skeletal muscle mass, it was chosen for all four assessments [36].

Appendicular skeletal muscle mass (ASM) was determined as the total skeletal muscle mass of the four limbs. This muscle mass is attached to the skeleton and plays a fundamental role in systemic movement and posture maintenance [17].

The upper and lower extremity muscle masses (arm + leg muscle mass [kg]) were summed and divided by height (meters) squared (m^2^) to calculate the skeletal muscle index (ASMMI) or the Baumgartner index (ASMMI = ASM/height^2^) [47, 48]. Sarcopenia assessment results vary among groups, but the ASMMI score is the most widely considered variable [15].

As recommended by the EWGSOP2, the following ASMM and ASSMI cutoff values were used in the present study: ASSM < 20 kg and ASSMI < 7.0 kg/m^2^ for males and ASSM < 15 kg and ASSMI < 5.5 kg/m^2^ for females [16]. As such, we propose the use of the Baumgartner index as a cutoff for ASMM/weight < 28.27% for males and < 23.47% for females. The FNIH uses another ratio, which is considered ASMM/BMI < 0.789 for males and < 0.512 for females.

#### Sarcopenia Severity Level—SEVERITY

##### Physical Performance:

Once the diagnosis of sarcopenia was established, the severity was assessed using the 400-m walk test, which assesses walking endurance and ability. During the test, participants were allowed to take up two rest breaks and were required to perform 20 laps of 20 m each as quickly as possible [49, 50]. When the test was not finished or took longer than 6 min to complete, the participants were classified as having low physical performance [15].

### The Algorithm for Diagnosing Sarcopenia and Sarcopenic Obesity

Sarcopenia according to the EWGSOP2 criteria: Based on low muscle strength, low muscle mass and low physical performance (male grip strength < 27 kg, ASMM < 20 kg, ASMMI < 7.0 kg/m^2^, and 400-m walk test ≥ 6 min; female grip strength < 16 kg, ASMM < 15 kg, ASMMI < 5.7 kg/m^2^, and 400 m walk test ≥ 6 min), sarcopenia was diagnosed and classified according to severity [15].

Sarcopenic obesity according to EASO/ESPEN and FNIH: The parameters BMI, waist circumference (WC), and ASMM score based on weight and BMI (BMI > 30 kg/m2, WC ≥ 102 cm for males, and ≥ 88 cm for females; ASMM/weight < 28.27% for males and < 23.47% for females; and ASMM/BMI < 0.789 for males and < 0.512 for females) were added to the EWGSOP-2 variables [17, 19].

### Intervention

Intervention Group: The intervention group took part in a structured exercise program aimed at enhancing muscle strength, endurance, and overall physical function. The exercise combined aerobic and strength training in the same session in a progressive circuit training program. All exercise program was previously described in our EXPOBAR protocol [27]. The exercise prescriptions included information about frequency, intensity, time, type, volume, and progression (FITT-VP) [20].

Based on the recommendations of the World Health Organization (WHO) [51], ASMBS statement [22], ACSM guidelines [20], and in our systematic review results [52], the duration of the program was 16 weeks, with a frequency of three times a week, for up to 55 min per session and starting one month after surgery. Each session started with a 5-min warm-up and finished with a 10-min cool-down, with stretching and flexibility work.

**Fig. 2:**
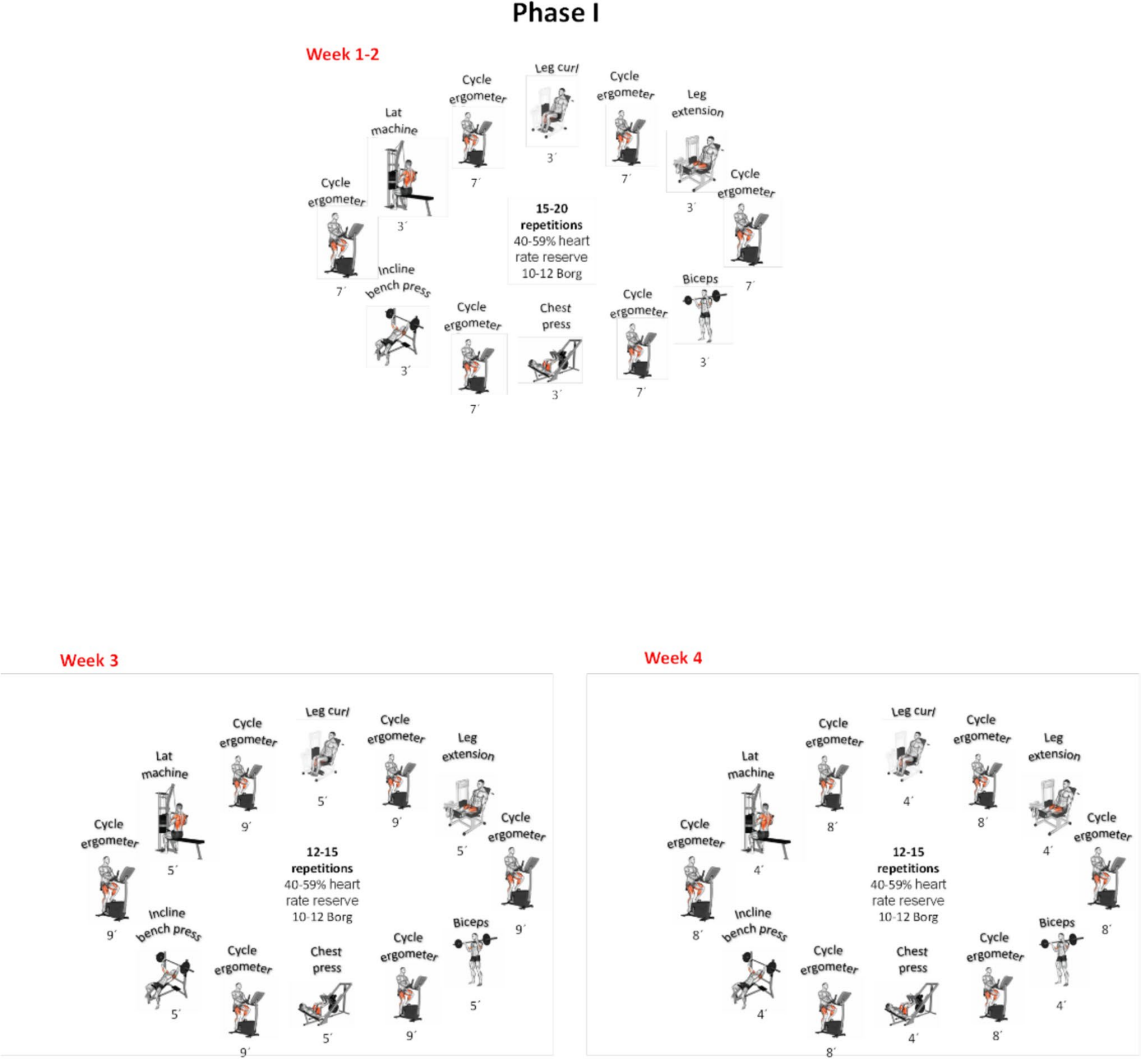

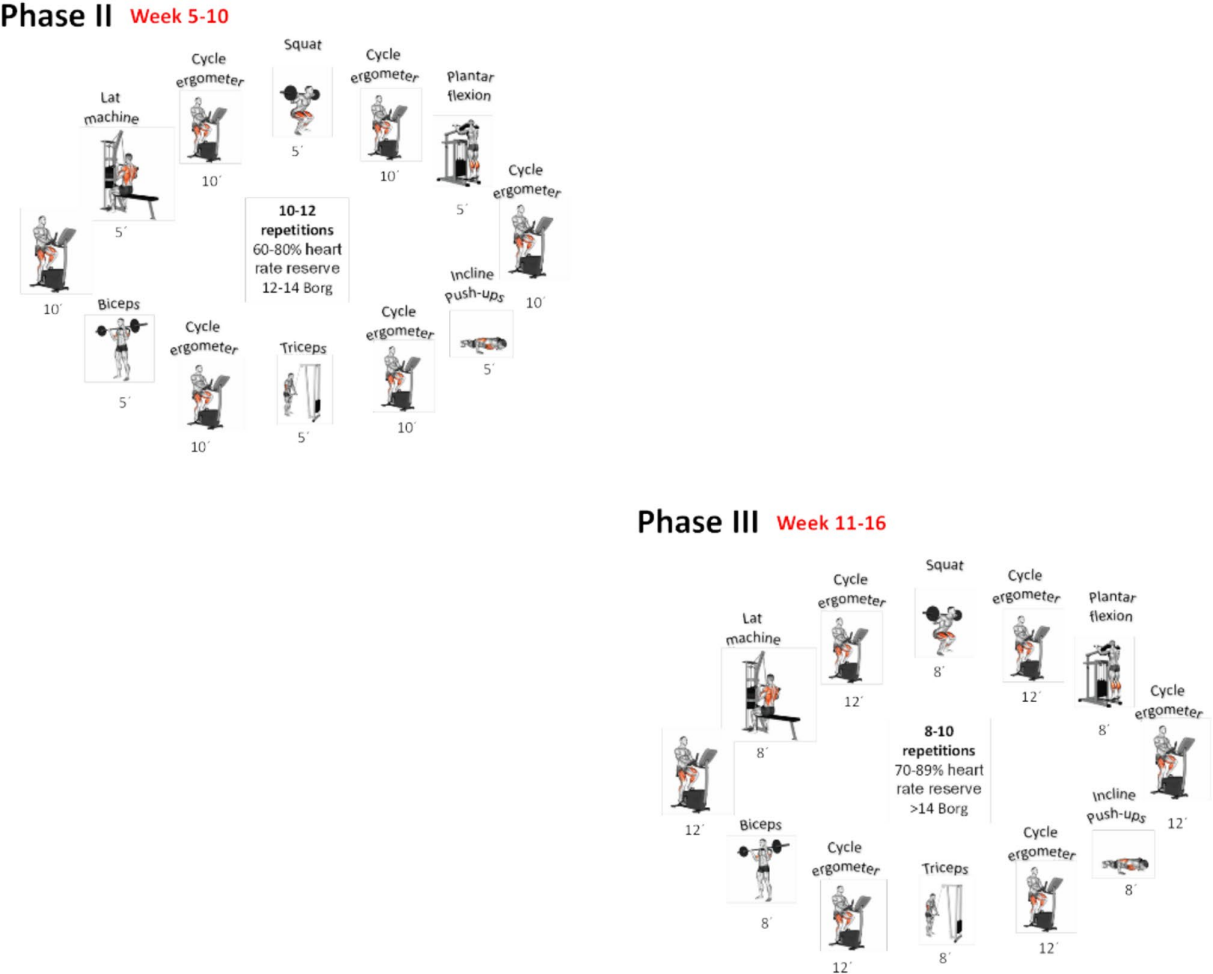
FITT-VP exercise for individuals after bariatric surgery

Patients assigned to the IG engaged in a circuit training program lasting for 16 weeks. Each exercise session included the following components: (a) a 5-min specific warm-up, (b) phase 1—resistance training (weeks 1–4), (c) phase 2—hypertrophy training (weeks 5–10), (d) phase 3—strength training (weeks 11–16), and a 10-min cool-down for flexibility (myofascial release, mobility, static, and dynamic stretching). The detailed exercise program is presented in Fig. 2.

The first phase included 20 min of interval training, encompassing circuit strength training. Each posterior phase has an increment of 10 min in the central block, always with an interval assessment of the patient’s adaptive response, based on heart rate reserve and the Borg scale [53].

Three personal trainers with training in sports sciences assessed physical fitness and prescribed and accompanied the training sessions. All training sessions took place in a fitness facility three times per week on nonconsecutive days from 7 to 10 a.m. The patients participated in the sessions in small groups (1–3) and were educated and motivated to exercise regularly.

Control Group: Participants in the control group received standard care, including regular health check-ups and nutritional counseling, but did not participate in any additional structured exercise program.

### Statistical Methods

The statistical analysis was performed with SPSS version 27.0 (IBM SPSS, Inc., Chicago, IL, USA) to determine the outcomes. To assess whether the incidence of risk and diagnosis of sarcopenia were dependent on exercise, the chi-square test was used, and a type I error probability of 0.05 was used for all the inferential analyses. Categorical variables are expressed as frequencies and percentages, and continuous variables are expressed as the mean and standard deviation. The percentages were compared using the chi-square test or Fisher’s exact test. Data normality was assessed with the Shapiro‒Wilk test, and group differences were examined with an independent t test or a Mann‒Whitney test. To compare dependent variables, two-way ANOVA with sphericity and homogeneity tests, Spearman correlation, and regression logistic analyses were performed considering the intervention group and control group and the four sequential assessments (one pre- and three post-intervention programs).

## Results

A total of 37 patients participated in this study. The mean age was 46.9 (± 11.4) years and the mean BMI was 42.9 (± 5.14) kg/m^2^. All patients were previously sedentary before surgery. Of the study participants, 15 patients (33.3%) had T2DM, 13 (28.8%) had arterial hypertension, 19 (42%) had dyslipidemia, 10 (22.2%) had hypothyroidism, and 17 (37.8%) had obstructive sleep syndrome apnea (OSAP).

The participants were randomly assigned to the IG or CG. At baseline, no significant differences were observed between the groups (*p* > 0 0.05), except for weight, which was greater in the IG (103 ± 14.5 V, 91.8 ± 14.7; *p* = 0.025), as presented in Table 1.

**Table 1:** Patient baseline characteristics in the intervention and control groups

Variables (mean ± SD)	Intervention group	Control group	*p* value
Age (years)	43.68 ± 11.00	50.53 ± 10.97	0.071
Anthropometry			
Weight (kg)	103 ± 14.5	91.8 ± 14.7	**0.025**
BMI (kg/m^2^)	37.8 ± 5.38	37 ± 4.53	0.639
Waist circumference (cm)	112 ± 9.68	110 ± 11.4	0.459
Body composition			
Fat mass (kg)	44.26 ± 12.16	44.47 ± 9.97	0.957
Body fat (%)	44.2 ± 6.46	45.5 ± 4.99	0.489
Total SMM mass (kg)	52.09 ± 9.05	48.01 ± 9.47	0.195
ASMM (kg)	22.29 ± 3.92	19.72 ± 4.79	0.086
ASMMI (kg)/m^2^)	8.09 ± 1.03	7.92 ± 1.47	0.672
ASMM/Weight (%)	21.7 ± 3.44	21.4 ± 3.18	0.795
ASMM/BMI	0.601 ± 0.14	0.535 ± 0.12	0.130
Physical function and strength			
Handgrip (kg)	22.4 ± 10.6	18.1 ± 6.44	0.154
30-s sit-to-stand test (s)	14.2 ± 2.35	12.8 ± 3.33	0.138
400-m walk test (m)	7.24 ± 2.26	8.79 ± 3.40	0.113

*BS*: Bariatric Surgery, *BMI*: Body Mass Index, *SMM*: Skeletal Muscle Mass, *ASMM*: Appendicular Skeletal Muscle Mass, *ASMMI*: Appendicular Skeletal Muscle Mass Index

Statistical significance was considered when *p-value* <0.05, highlight in bold

To determine the prevalence of sarcopenia and sarcopenic obesity, three algorithms were used to assess the parameters of these diseases (Table 2).

**Table 2:** Main outcomes of the EXPOBAR trial

Variables (mean ± SE)		Before exercise	After exercise			Global group effect		Intervention effect Moment * group							
Moment		Baseline T0	4-month T1	6-month T2	12-month T3	p value	Effect size (*η*^2^)	T0 vs T1		T1 vs T2		T2 vs T3		T0 vs T3	
	Group							*p* value	Effect size (*η*^2^)	*p* value	Effect size (*η*^2^)	*p* value	Effect size (*η*^2^)	*p* value	Effect size (*η*^2^)
Anthropometry															
Weight (kg)	**IG** **CG**	103.2 ± 14.5 91.8 ± 14.7	83.1 ± 10.9^a^ 75.4 ± 14.7^a^	75 ± 9.2^b^ 69.9 ± 12.1^b^	74 ± 9.9^c,e^ 70.8 ± 12.0^c,e^	**0.039**	0.078^#^	0.137	0.064^#^	0.280	0.034^#^	0.191	0.050	**0.048**	0.111^**#**^
BMI (kg/m^2^)	**IG** **CG**	37.8 ± 5.40 37 ± 4.53	30.4 ± 4.43^a^ 30.2 ± 4.17^a^	27.5 ± 4.01^b^ 28.2 ± 4.34	27.1 ± 3.8^c,e^ 28.7 ± 4.93^c,e^	0.142	0.052^+^	0.531	0.012^+^	0.307	0.031^+^	0.112	0.072^#^	0.106	0.075^#^
Waist circumference (cm)	**IG** **CG**	112.2 ± 9.68 109.5 ± 11.4	97.2 ± 8.4^a^ 97.2 ± 12.8^a^	90.5 ± 8.49^b^ 94.2 ± 12.2	88.2 ± 8.8^e^ 93.6 ± 11^e^	**0.010**	0.105*	0.316	0.030^+^	**0.027**	0.136^*^	0.292	0.033^+^	**0.023**	0.143*
Total weight loss (%)	**IG** **CG**	12.8 ± 2.72 13.1 ± 4.32	29.5 ± 5.92^a^ 29 ± 4.58^a^	36.1 ± 7.5^b^ 33.8 ± 7.17^b^	37 ± 8.08^c,e^ 32.5 ± 9.76^c,e^	0.161	0.049^+^	0.675	0.005^+^	0.336	0.027^+^	0.111	0.073^+^	0.106	**0.075**^**#**^
Body composition															
Fat mass (kg)	**IG** **CG**	44.26 ± 12.16 44.47 ± 9.97	27.66 ± 13.55^a^ 34.42 ± 7.92^a^	25.22 ± 9.14 34.19 ± 6.99	23.37 ± 9.45^e^ 30.45 ± 12.6^e^	**0.006**	0.114*	**0.001**	0.272*	0.744	0.003^+^	0.521	0.012^+^	0.052	0.107^#^
Body fat (%)	**IG** **CG**	44.2 ± 6.42 45.5 ± 4.99	36.6 ± 2.29^a^ 40.6 ± 7.26^a^	31.9 ± 7.96^b^ 37.4 ± 6.34^b^	30.4 ± 6.22^c,e^ 37 ± 7.71^e^	**0.044**	0.076^#^	0.095	0.080^#^	0.200	0.048^#^	0.650	0.006^+^	**0.010**	0.180*
Total SMM mass (kg)	**IG** **CG**	52.10 ± 9.04 48.01 ± 9.47	45.43 ± 13.2^a^ 44.65 ± 9.54^a^	44.53 ± 9.59 39.99 ± 5.08^b^	37.1 ± 12.7^c,d,e^ 37.14 ± 5.06^c,e^	0.138	0.052^+^	0.169	0.055^+^	0.133	0.065^#^	**0.003**	0.233*	0.219	0.044^+^
ASMM (kg)	**IG** **CG**	22.29 ± 3.92 19.72 ± 4.79	20.45 ± 3.71^a^ 17.53 ± 4.11^a^	18.94 ± 4.14^b^ 15.91 ± 4.06^b^	17.02 ± 3.8^c,d,e^ 13.86 ± 4.31^c,d,e^	0.705	0.014^+^	0.386	0.022^+^	0.817	0.002^+^	0.766	0.003^+^	0.362	0.024^+^
ASMMI (kg)/m^2^)	**IG** **CG**	8.09 ± 1.03 7.92 ± 1.48	7.43 ± 0.99^a^ 7.03 ± 1.22^a^	6.85 ± 1,20^b^ 6.39 ± 1.28^b^	6.17 ± 1.05^c,d,e^ 6.17 ± 1.05^c,d,e^	0.186	0.046^+^	0.163	0.056^+^	0.718	0.004^+^	0.395	0.021^+^	0.078	0.088^**#**^
ASMM/weight (%)	**IG** **CG**	21.7 ± 3.44 21.4 ± 3.18	24.08 ± 4.35^a^ 23.3 ± 3.13^a^	25.3 ± 4.68 22.8 ± 3.71	22.9 ± 3.39^d^ 19.8 ± 5.82^c,d^	**0.030**	0.084^#^	0.129	0.066^#^	0.237	0.041^#^	0.476	0.015^+^	**0.014**	0.164*
ASMM/BMI	**IG** **CG**	0.601 ± 0.14 0.535 ± 0.12	0.687 ± 0.17^a^ 0.580 ± 0.11	0.701 ± 0.18 0.570 ± 0.14	0.635 ± 0.1^d^ 0.496 ± 0.19^c,d^	**0.041**	0.077^#^	0.063	0.098^#^	0.286	0.033^+^	0.781	0.002^+^	**0.020**	0.150*
Physical function and strength															
Handgrip (kg)	**IG** **CG**	22.4 ± 10.62 18.1 ± 6.44	24.8 ± 8.44 16.8 ± 5.25	26.9 ± 7.51 17.7 ± 5.11	27.4 ± 7.21 17.3 ± 5.44^e^	**0.005**	0.120*	**0.025**	0.140*	0.288	0.033^**+**^	0.590	0.009^+^	**0.014**	0.166*
Sit-to-stand test (s)	**IG** **CG**	14.2 ± 2.35 12.8 ± 3.33	15.9 ± 3.3^a^ 13.2 ± 2.88	16.5 ± 2.17 13.1 ± 2.45	16.8 ± 2.41^e^ 12.8 ± 2.70	** < 0.001**	0.162*	**0.040**	0.118*	0.323	0.029^+^	0.073	0.092^#^	** < 0.001**	0.319*
400-m walk test (m)	**IG** **CG**	7.24 ± 2.26 8.79 ± 3.40	6.05 ± 1.86^a^ 8.74 ± 3.05	5.82 ± 1.58 9.58 ± 3.58^c^	5.57 ± 1.19^**e**^ 10.14 ± 4.1^c,d^	** < 0.001**	0.303*	**0.021**	0.146*	**0.007**	0.193*	**0.033**	0.126*	** < 0.001**	0.424*

ANOVA was used to assess the global group effect at all time points. The post hoc test revealed results within each group; a *p* value <0.05 indicated statistical significance, highlight in bold

*BMI* body mass index, *SMM* skeletal muscle mass, *ASMM* appendicular skeletal muscle mass, *ASMMI* appendicular skeletal muscle mass index

^a^T0 vs T1 post hoc test, Bonferroni significance; ^b^T1 vs T2 post hoc test, Bonferroni significance; ^c^T1 vs T3 post hoc test, Bonferroni significance; ^d^T2 vs T3 post hoc test, Bonferroni significance; ^e^T0 vs T3 post hoc test, Bonferroni correction, significant

^+^Small effect size (0.01-0.06); ^#^ Medium effect size (0.06-0.14); * Large effect size (>0.14)

The EXPOBAR trial showed that the intervention had significant and meaningful effects on several anthropometric measures, body composition parameters, physical function, and strength outcomes. While several variables, such as BMI and ASMM score, had nonsignificant global group effects (*p* = 0.142, *p* = 0.705), they still had significant effects at 4, 6, and 12 months according to the post hoc test (*p* < 0.05). However, muscle mass decreased continuously after surgery in both groups despite the exercise protocol used in the intervention group.

The most substantial improvements were observed in physical function and strength metrics, suggesting that the intervention was effective at enhancing physical fitness, with a significant group effect (*p* < 0.001) and large effect size (*η*^2^ = 0.303). Additionally, handgrip strength and lower body strength had significant group effects (*p* = 0.005 and *p* < 0.001, respectively) and large effect sizes (*η*^2^ = 0.120 and *η*^2^ = 0.162). There were notable and meaningful increases in the measured outcomes over all interval periods, and these increases were substantial enough to have practical benefits or applications (Table 2).

The latest and most important guidelines about this subject were used. The EWGSOP2 group consensus used the FACS sequence assessment, while the EASO/ESPEN and FNIH algorithms were based on weight and BMI criteria (Table 3).

**Table 3:** Logistic regression analysis of the ability of different *algorithms to diagnose sarcopenia – EASO, EWGSOP2, FNIH*

	Intervention group				Control group				Intervention effect vs baseline		
Major risk and severity (%)	Pre-exercise	After exercise			Pre-exercise	After exercise			*p* value		
	Baseline T0	4-month T1	6-month T2	12-month T3	Baseline T0	4-month T1	6-month T2	12-month T3	4-month	6-month	12-month
EASO											
*SCREENING*—High BMI	100%	31.6%	31.6%	26.3%	100%	35.3%	35.3%	41.2%	0.892	0.629	0.280
*SCREENING*—High Waist circumference	100%	73.7%	42.1%	10.5%	100%	82.4%	58.8%	58.8%	0.995	0.293	0.113
*SCREENING*—SARC-F	84.2%	5.3%	10.5%	0%	88.2%	70.6%	58.8%	0%	** < 0.001**	**0.005**	1.000
*DIAGNOSIS*—Altered functional skeletal muscle	47.4%	15.8%	5.3%	10.5%	70.6%	64.7%	52.9%	52.9%	**0.005**	**0.008**	**0.011**
*DIAGNOSIS –*Altered body composition	89.5%	42.1%	52.6%	84.2%	88.2%	70.6%	88.2%	94.1%	0.179	0.464	0.728
*STAGING –* STAGE II: With complications	100%	15.8%	5.3%	10.5%	100%	100%	100%	100%	**0.034**	**0.002**	** < 0.001**
EWGSOP2											
*SCREENING*—SARC-F	84.2%	5.3%	10.5%	0%	88.2%	70.6%	58.8%	0%	** < 0.001**	**0.005**	1.000
*ASSESS*—Skeletal muscle strength	47.4%	15.8%	5.3%	10.5%	70.6%	64.7%	52.9%	52.9%	**0.005**	**0.008**	**0.011**
*CONFIRM*—Skeletal muscle mass	0%	0%	15.8%	47.4%	0%	11.8%	29.4%	52.9%	0.077	0.325	0.294
*SEVERITY*—Physical performance	73,7%	42.1%	31.6%	21.1%	70.6%	76.5%	82.4%	94.1%	**0.034**	**0.002**	** < 0.001**
FNIH											
*SKELETAL MUSCLE STRENGTH*	47.4%	15.8%	5.3%	10.5%	70.6%	64.7%	52.9%	52.9%	**0.005**	**0.008**	**0.011**
SKELETAL MUSCLE MASS	47.4%	21.1%	15.8%	26.3%	64.7%	23.5%	47.1%	76.5%	0.858	**0.050**	**0.004**

The intervention effect was reported to the baseline. A *p* value <0.05 was considered to indicate statistical significance, highlight in bold

*BMI* Body mass index

The first step in all the algorithms is screening. The FACS uses the SARC-F questionnaire to determine risk. The EASO and ESPEN use both BMI and waist circumference. Before the exercise program, all patients met these two last criteria as positive criteria. Immediately after the exercise program, the patients presented different screening results (IG 5.3% *versus* CG 70.6%, *p* < 0.001), with a decreased risk in the IG after the exercise intervention. The same result was obtained at six months (10.5% for the IG *versus* 58.8% for the CG; *p* = 0.005).

According to the results of assessing, confirming, and diagnosing sarcopenia, there was a decrease after the exercise intervention in the IG (47.4% to 15.8%) and a small difference in the CG (70.6 to 64.7%). This difference was significant after the exercise program (*p* = 0.005), at 6 months (*p* = 0.008) and 12 months (*p* = 0.011). To confirm the diagnosis, three different indices and cut-offs were used to assess muscle mass.

The ASMMI did not significantly differ between the groups. When the EASO/ESPEN criteria were applied based on weight, the results were similar. Different results were found for the FNIH criteria, which did not significantly improve after the exercise intervention (IG 21.1% *versus* CG 23.5%) but did improve long-term after exercise at 6 (IG 15.8% *versus* CG 41.7%) and 12 months (IG 26.3% *versus* CG 76.5%).

The severity of sarcopenia was significantly different immediately after and long after the exercise program (*p* = 0.034, *p* = 0.002, *p* < 0.001).

To perform a more detailed analysis of the results associated with physical exercise and its temporal impact on the risk and incidence of sarcopenia, logistic regression was carried out to analyze the causal relationship in addition to the associations already described. There was a greater number of participants at risk of sarcopenia in the CG than in the control group (5.3% *versus* 70.6%, respectively; *p* < 0 0.001). This effect was maintained at 6 months (*p* = 0.005) but not at 12 months (*p* > 0.05).

Regarding sarcopenia screening and diagnosis, 64.7% of the CGs had a positive assessment for subsequent diagnosis of sarcopenia after exercise (*p* = 0.005). In comparison, only 15.8% of the IG had the same positive assessment, highlighting the decrease in upper limb skeletal muscle strength in the CG. This relationship was maintained at 6 and 12 months (*p* = 0.008, *p* = 0.011). According to the results of the inferential statistical analysis, after exercise, there was a significant probability of confirming the diagnosis and changing the assessment of the level of sarcopenia between the IG and CG (*p* = 0 0.002).

There were no significant differences in the EASO or EWGSOP2 score, but when the FNIH consensus was used (ASMM/BMI), the differences in the confirmation of sarcopenia were statistically significant at 6 months (*p* = 0.05) and 12 months (*p* = 0.004).

## Discussion

The EXPOBAR trial is the first randomized controlled trial in Portugal to evaluate the effects of supervised and structured physical exercise programs on the risk and diagnosis of sarcopenia induced by bariatric surgery using the tools and cut-offs recommended by current consensuses.

Conflicting findings have been reported in several studies and papers regarding the impact of weight loss on sarcopenia and muscle deficiency. The clinical and biological effects of metabolic/bariatric surgery on sarcopenia and muscle deficiency are still not fully understood.

The current findings reveal a significant increase in the risk of sarcopenia within the first month after surgery. This can occur after bariatric surgery, particularly because of the very marked initial weight loss [54]. These alterations make it increasingly important to assess the risk of sarcopenia in patients undergoing bariatric surgery. The intense weight loss that occurs in the first few months [4] can lead to an increase in falls and fractures [55, 56] due to changes in proprioception [57] and the metabolic impact associated with bariatric surgery and obesity. These changes are closely associated with frailty and instability, especially, but not exclusively, among elderly people. [58, 59].

The present data show that the risk of initial sarcopenia is important and that the risk of sarcopenia can be clinically assessed and managed with early intervention. Based on the evidence, if a patient is at risk of sarcopenia and has been diagnosed with sarcopenia, treatment measures for the disease should be taken [60]. The first treatment option for sarcopenic obesity is to combine nutritional and exercise goals with the aim of reducing adipose tissue but also enhancing and preserving muscle mass and function [61]. Due to the significantly increased risk observed in the first month after surgery, prehabilitation and early interventions after surgery should probably be implemented.

In the absence of a gold standard, several different screening tools, strength tests, and skeletal muscle mass indices have been proposed for assessing sarcopenia. These indices consider adjustment factors such as height squared, weight, or BMI. By using these indices, healthcare professionals can better evaluate and diagnose sarcopenia [62]. In this study, these indices were used to diagnose sarcopenia and sarcopenic obesity, revealing that while some body composition metrics did not show significant group effects, the improvements in muscle function and strength underscore the importance of using objective diagnostic criteria.

The intervention group experienced significant improvements in muscle function and physical performance when compared with the control group, which emphasizes the potential of structured exercise programs to counteract the negative impacts of body composition on individuals associated with obesity, sarcopenia and bariatric surgery [11].

Bariatric surgery has an impact on adipose tissue, but in the first few months, it also has a relevant effect on muscle mass [63–65]. This highlights the importance of introducing circuit training (aerobic and strength training), as was the case in the present study, where an important positive impact of exercise on muscle quality and consequently a reduction in the risk of worsening and developing sarcopenia was found.

The ACSM recommends resistance exercise to improve the function of the musculoskeletal system [20]. Combined with aerobic training, it can potentially improve cardiorespiratory promotion of anabolic muscle adaptation and consequently improve muscle quality and quantity [53, 66, 67]. There was a difference in the evolution of these diagnostic criteria among the patients who practiced circuit training, as reported in a systematic review [68], but this difference was not statistically significant.

Major body composition metrics, BMI, and the ASMMI score were not significantly different among intervention and control groups, raising questions about the overall effectiveness of the intervention. Nevertheless, these parameters might not have been sensitive enough to detect differences between the two groups in the context of the strong confounding effects directly caused by the surgical procedure.

These findings underline an important point regarding the FITT-VP principle recommended by the ACSM and reported in our systematic review [26, 52, 69]. The lack of differences in the results shows that adjusting at least one of the exercise parameters, namely, frequency or intensity, may be necessary to obtain better results. This can be done in accordance with the ASMBS guidelines by increasing the training frequency.

Nevertheless, our results showed that circuit training significantly improved functional physical capacity and strength after the initial decline caused by bariatric surgery (these results were previously published) [4, 70–72]. In other words, exercise was able to reverse the functional and strength loss that was the result of the surgical procedure, with a reduction in the number of patients at risk of a diagnosis of sarcopenia after bariatric surgery. This finding aligns with previous research [73] indicating that weight loss can improve physical performance by reducing mechanical factors and improving mobility and walking time, although it was observed that there was a decrease in strength assessed by handgrip strength in the first year after bariatric surgery.

The study’s medium-term effects, particularly at 6 and 12 months, underscore the sustainability of improvements in muscle function and physical performance. At the 6-month mark, improvements in muscle function were evident, demonstrating that the initial gains were not short-lived. By 12 months, participants continued to exhibit enhanced physical performance, indicating that these benefits can be sustained with consistent exercise. These findings emphasize the critical importance of continuous exercise for maintaining and building upon the benefits observed. These sustained improvements suggest that regular exercise is essential not only for achieving initial gains but also for preserving and enhancing muscle function and physical performance over time.

However, there was muscle mass loss in both groups, and this loss continued throughout the study duration, despite the exercise protocol used in the intervention group.

In the present study, when the complete diagnostic criteria defined by EASO/ESPEN and EWGSOP2 were used, no significant differences were found in either index, which may indicate that it might be essential to use the muscle strength criterion, which is probably the most important and decisive criterion for diagnosing sarcopenia in patients undergoing bariatric surgery.

The fundamental component and first step in defining the diagnosis of sarcopenia is muscle function. In a clinical context, the sarcopenia diagnostic index associated with weight is an essential parameter that significantly affects the outcome after long-term exercise, as is the case with the adjustment for BMI suggested by the FNIH [17].

Changes in sarcopenia parameters, such as physical function, are associated with changes in muscle mass and overall body weight, suggesting that BMI is a valuable parameter for adjusting the risk of sarcopenia. Physical and functional capacity are important indicators for diagnosing muscle quality, and they are noninvasive indicators. In studies involving individuals with other medical conditions and healthy individuals, it was already observed that muscle quality measured by the handgrip test is a more reliable indicator than the quantity of the muscle itself [74–77].

Sarcopenia is a particularly concerning problem in the context of bariatric surgery because it can impair physical function, increase the risk of frailty, and negatively impact overall health outcomes. Sarcopenia is a complex and multifactorial condition that is influenced by a variety of factors, including age, physical activity, nutritional status, and underlying medical conditions. Accurately defining sarcopenia parameters after bariatric surgery patients is therefore crucial for the effective management and prevention of this condition.

Muscle strength, a variable not directly impacted by measurements of weight loss, might be a more useful indicator of sarcopenia in bariatric patients, whereas both fat and muscle mass are heavily influenced and confounded directly by weight loss.

## Conclusion

Sarcopenia, characterized by a loss of muscle mass and function, is a growing concern in various populations, including those undergoing bariatric surgery. This study demonstrated significant improvements in the risk of sarcopenia, muscle quality, and functional capacity following a 16-week supervised circuit training program. However, the changes in muscle quantity were not statistically significant. These findings highlight the importance of incorporating exercise interventions, as recommended by the ACSM and the ASMBS, with an emphasis on increasing the frequency and intensity of training to combat sarcopenia effectively.

Management of sarcopenia requires regular monitoring of both body composition and muscle function. Assessments of strength, skeletal muscle mass, and functional status, along with periodic monitoring of body fat composition, are essential for tracking progress and optimizing treatment strategies over time. By going beyond BMI and addressing other parameters, such as physical function, healthcare providers can more accurately identify potential sarcopenia development after bariatric surgery, allowing appropriate intervention design.

Individualized care plans that consider the unique needs and circumstances of patients are crucial for achieving positive clinical outcomes. Preventive combined exercise programs are particularly important for patients undergoing bariatric surgery, as they can significantly improve strength and metabolic health.

Further research and clinical trials are warranted to refine and expand the current approaches available for managing sarcopenic obesity. Such efforts should aim to improve the quality of life and clinical outcomes for individuals affected by this complex condition, ensuring that interventions are both effective and sustainable over the long term.

## Data Availability

Corresponding author had full access to all the data and participants data are available upon request for authors. The authors have the responsibility for mantenance the integrity of anonymized format available from request.
